# The chain mediating effects of resilience and perceived social support in the relationship between perceived stress and depression in patients with COVID-19

**DOI:** 10.3389/fpsyg.2024.1400267

**Published:** 2024-08-29

**Authors:** Lingling Wang, Jing Yu, Xuqian Diao, Yuanbei Zhang, Ye Miao, Wei He

**Affiliations:** ^1^Department of Military Medical Psychology, Air Force Medical University, Xi’an, China; ^2^Department of Radiology, 986 Hospital of Air Force Medical University, Xi’an, China; ^3^Department of Obstetrics and Gynaecology, Tangdu Hospital of Air Force Medical University, Xi'an, China; ^4^Department of Radiation Protection Medicine, Faculty of Preventive Medicine, Ministry of Education Key Lab of Hazard Assessment and Control in Special Operational Environment, Air Force Medical University (Fourth Military Medical University), Xi’an, China

**Keywords:** resilience, depression, perceived social support, perceived stress, COVID-19 patients, chain mediating effect

## Abstract

**Introduction:**

Perceived stress and depression were indirect effects of the COVID-19 pandemic, especially in square-cabin hospitals. It was paramount to understand their mediating effects, which might detonate factors that led to mental illness.

**Materials and methods:**

We conducted a cross-sectional study to investigate perceived stress and depressive symptoms among patients with COVID-19 in Shanghai square-cabin hospitals from April 18 to May 19, 2022. The questionnaire included the Perceived Stress Scale 10, Patient Health Questionnaire 9, Perceived Social Support Scale, and the Connor-Davidson Resilience Scale 10.

**Results:**

This study investigated the chain-mediating roles of perceived social support and resilience in the relationship between perceived stress and depression. Perceived stress positively predicted depression (*r* = 0.613, *p* < 0.01), negatively correlated with perceived social support (*r* = −0.318, *p* < 0.01) and resilience (*r* = −0.398, *p* < 0.01). In the chain mediating model, perceived stress had significant direct predictive effects on depression, and significant indirect predictive effects on depression through perceived social support and/or resilience.

**Conclusion:**

It showed that higher perceived social support and resilience were associated with lower perceived stress among COVID-19 patients, which might lead to symptoms of mild depression, and highlights the importance of resilience and perceived social support in reducing depressive symptoms.

## 1 Introduction

Coronavirus disease 2019 (COVID-19) has become a public health threat worldwide. In this situation, a series of objective events triggered by the pandemic have led to different levels of psychological problems in different populations. The COVID-19 patients suffer from anxiety, stress and restlessness (Duan and Zhu, 2020; Matalon et al., 2021; Robinson et al., 2022). In addition to the direct effects of COVID-19, the pandemic has created an environment in which many determinants of mental health are also affected (Damian et al., 2021). The feeling of experiencing stress may also be aggravated by centralized isolation and treatment, dense population, unfamiliar environment, cohabitation of COVID-19 patients, depressing environment of the ward, family members and loved ones suffering from COVID-19 or worsening of the disease or death due to COVID-19 (Awano et al., 2020; Lu et al., 2021). Therefore, they might be considered as one of the most vulnerable people to experience psychological stress and other mental health symptoms (Du et al., 2024).

The COVID-19 patients were reported high stress levels during the COVID-19 epidemics (Vindegaard and Benros, 2020; Matalon et al., 2021; Fu et al., 2022; Malinauskas and Malinauskiene, 2022). Perceived stress is an individual response or experience to a stimulus, threat or event in different circumstances, which is one of the gateways to several mental health disorders, such as anxiety, depression, phobia, post traumatic disorder and even psychotic disorders (Banjongrewadee et al., 2020). Numerous research has suggested a link between perceived stress and depressive symptoms (Spada et al., 2008; Besharart et al., 2020; Yan et al., 2021; Dymecka et al., 2022; Lathabhavan and Vispute, 2022). The fear of disease and the pathological state of the body could aggravate the perceived stress symptoms of COVID-19 patients, which is one of the important reasons for the depression (Li J. et al., 2020; Li Z. et al., 2020; Lu et al., 2021). However, the internal mechanism of the relationship between perceived stress and depression during the COVID-19 pandemic remain largely unexplored (Duan and Zhu, 2020; Damico et al., 2021). Models of the stress-depression relationship emphasized the importance of perceived stress and coping resources (Hewitt et al., 1992; Dulaney et al., 2008). Previous studies have paid more attention to how perceived stress negatively affect patients, and generally ignored the protective or buffering factors on depression (Banjongrewadee et al., 2020; Dymecka et al., 2022; Hu et al., 2022). Therefore, in order to better respond to mental health problems, we need to identify the protective or buffer factors for the COVID-19 patients, focusing on people’s positive psychological strength from a new perspective.

Among several protective factors, social support and psychological resilience have been proven to alleviate the negative effects of stress and depression (Santini et al., 2015; Bloom et al., 2017; Labrague et al., 2018; Chen and Bonanno, 2020). Social support improved mental status possibly in the form of benefits from social relationships and a buffer against stressful situations (Alsubaie et al., 2019). One study has pointed that social support would be a potential determinant for depression during the COVID-19 pandemic (Chan and Lee, 2022). Perceived social support is considered to be one of the important social factors for predicting individual depression (Hyde et al., 2011). There is a direct or indirect relationship between perceived social support and depression (Cohen and Wills, 1985). Previous studies found that higher level of perceived social support is associated with lower level of depression (Grey et al., 2020; Chan and Lee, 2022). Some studies have shown that positive experiences of social support foster resilience to stress and prevent psychopathology (Ozbay et al., 2007). In addition, the predictive effect of social support on mental health can be achieved through resilience (Wilks and Croom, 2008).

As a key psychosocial factor, resilience can help individuals cope with stress more effectively when they experience psychological pain in stressful situations (Richardson, 2002). Studies indicated that resilience was a protective factor for COVID-19 patients and was negatively correlated with depression (Awano et al., 2020), people with high resilience were less likely to suffer from depression (Benedek et al., 2007). Some studies pointed out that resilience worked through interaction with the environment (Luthar et al., 2000). As an external environmental factor, social support enhanced individuals’ adaptability to adverse conditions by endowing and mobilizing their psychological resources, thus improving their mental health status (Chen, 2019).

This study aimed to elucidate the mechanisms underlying the associations between stress, perceived social support, resilience, and depression in patients with COVID-19. Patients with COVID-19 isolated from Shanghai Makeshift Hospitals were enrolled in this study. We not only analyzed the independent mediating effects of perceived social support and mental resilience, but also analyzed the chain mediating effects of perceived social support and mental resilience. This may provide a better understanding of the stressful events experienced by patients and their contribution to the risk of depression. We speculated that the association between stress and depressive symptoms is mediated by perceived social support and mental resilience. Specifically, we hypothesized that individuals in stressful situations have lower levels of perceived social support and mental resilience, which may further lead to more severe depressive symptoms. Furthermore, we predicted that the effect of psychological resilience on depression in stressful situations is influenced by perceived social support, that is, high levels of perceived social support would strengthen the association between stress, resilience, and depressive symptoms.

## 2 Materials and methods

### 2.1 Participants

The participants were COVID-19 patients at Shanghai square-cabin hospitals from April 18 to May 19, 2022. Considering data collection and patient isolation, an online method was used. They completed the questionnaires on their mobile phones by scanning a QR code which were provided by an online platform.^1^ All participants gave their electronic informed consent after understanding the research content and the rights and obligations of the researcher and participants. A total of 1,014 questionnaires were collected, of which 126 invalid questionnaires were excluded. The exclusion criteria were as follows: (1) the time took less than 100 s to complete the survey; (2) selection of the same option for all items; (3) the total score exceeded three standard deviations. The remaining 888 valid questionnaires constituted the data for this study. The effective utilization rate was 87.57%.

### 2.2 Measures

#### 2.2.1 Perceived stress scale questionnaire (PSS-10)

Perceived stress symptoms were assessed using the Simplified Chinese Perceived Stress Scale (PSS-10) questionnaire (Lee and Crockett, 1994), based on the PSS-10 (Cohen et al., 1983). The PSS-10 includes 10 items, each rated on a 5-point scale from 0 (never) to 4 (very common). Higher scores indicate higher levels of perceived stress. The scale has been demonstrated with strong reliability and validity (Sun et al., 2019; Hu et al., 2023). In the current study, the Cronbach’s α coefficient for the entire scale was 0.861.

#### 2.2.2 Patient health questionnaire (PHQ-9)

Depression was measured using the validated Chinese version of the 9-item self-report Patient Health Questionnaire-9 (PHQ-9) (Yu et al., 2012), based on the nine Diagnostic and Statistical Manual of Mental Disorders-IV (DSM-IV) criteria which was employed to assess the frequency of depressive symptoms over the past 2 weeks (Spitzer, 1999). The Patient Health Questionnaire-9 (PHQ-9) is a widely used self-rated version to ascertain the mental state of COVID-19 patients (Liu C. H. et al., 2020; Liu J. et al., 2020; Cha et al., 2022; Zhao et al., 2022). The score for each item varies from 0 (not at all) to 3 (nearly every day). The sum of the scores ranges from 0 to 27, and a higher total PHQ-9 score indicates more severe depressive symptoms. In this study, the Cronbach’s α coefficient for the entire scale was 0.911.

#### 2.2.3 Perceived social support scale (PSSS)

The Perceived Social Support Scale (PSSS) is a 12-item self-report measure in which participants indicate their perception of support from friends, family, and a significant other on a seven-point Likert scale ranging from 1 (very strongly disagree) to 7 (very strongly agree) (Zimet et al., 1988). The modified Chinese PSSS validated by Wang was used in this study (Wang et al., 1999). The scale has been used with excellent psychometric properties (Wen et al., 2020; Zhao et al., 2023). In this study, the Cronbach’s α coefficient for the entire scale was 0.961.

#### 2.2.4 Connor-Davidson resilience scale (CD-RISC 10)

The 10-item Connor-Davidson Resilience Scale (CD-RISC 10) is a common scale used to assess resilience (Campbell-Sills and Stein, 2007). The translated Chinese version was used in this study (Lyu et al., 2017). The CD-RISC 10 includes 10 items, each rated on a 5-point scale from 0 (never) to 4 (very common). Previous studies have shown that this scale was in excellent reliability and validity (Fu and Wang, 2022; Lu et al., 2024). Higher scores on this scale are interpreted as higher resilience. In this study, the Cronbach’s α coefficient for the entire scale was 0.956.

### 2.3 Statistical analyses

**Figure 1:**
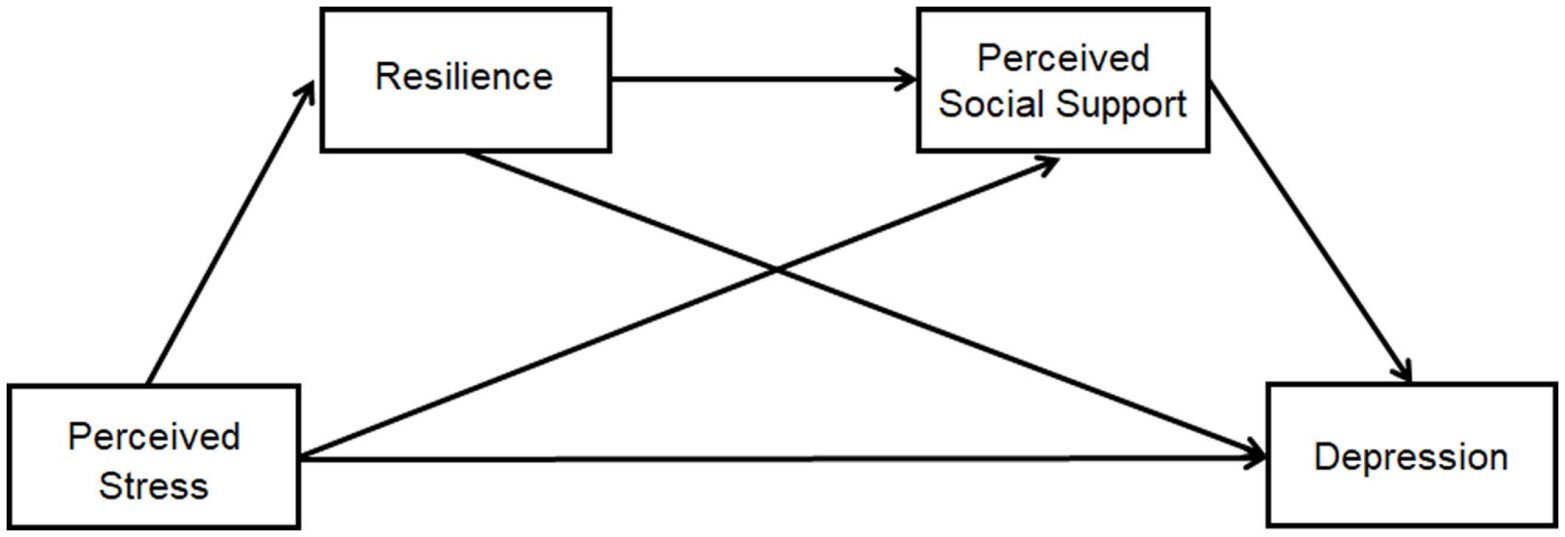
Conceptual chained mediation model. The prediction of a chain mediating model of resilience and perceived social support on the relationship between perceived stress and depression among patients with COVID-19 in Shanghai square-cabin hospitals.

IBM’s SPSS software (version26.0) was used to analyze the data. The results of common method bias were examined using the Harman one-way test (Podsakoff et al., 2003), which indicates a serious common bias if one factor explains more than 50% of the common variance (the result is 40.98%) (Podsakoff and Management, 1986). Measurement data conforming to a normal distribution were expressed as mean ± SD, and count data were described using frequency and composition ratios. Bivariate relationships between the variables were determined using Pearson’s correlation analysis. The bias-corrected percentile bootstrap method was employed to test the hypothesized chain mediation model. Chain-mediated effect implementation (Model 6, Process Macro) (Hayes, 2015) was performed by repeated sampling 5,000 times. The 95% corrected confidence interval (CI) method-mediated effects were considered statistically significant if the 95% bootstrap CI for the indirect effect did not contain zero (Preacher and Hayes, 2008). The direct effect was considered when the independent variable directly affected the dependent variable, and when the 95% CI of the bootstrap test did not include zero, the effect was valid. An indirect effect was considered when the independent variable had an effect on the dependent variable through the intermediary variable, and when the 95% CI did not include zero via the bootstrap method, the indirect effect was valid. Figure 1 shows the conceptual chained mediation model of the current study.

## 3 Results

### 3.1 Descriptive analysis

A total of 888 patients with valid questionnaires in square-cabin hospitals in Shanghai were analyzed in this study, of whom there were 517 males (58.22%) and 371 were females (41.78%). All of them had tested positive for COVID-19 and received the corresponding treatment for COVID-19. Means, frequencies and standard deviations (SDs) were used to quantify the data. Patient variables were analyzed using the independent sample t-test for gender characteristic and using analysis of variance for age, education and job characteristics. There were differences in the questionnaire scores of the Perceived Stress Scale 10 (PSS-10), Patient Health Questionnaire 9 (PHQ-9), Perceived Social Support Scale (PSSS), and Connor-Davidson Resilience Scale10 (CD-RISC10) between the sexes. Women demonstrated higher scores in Perceived Stress and Patient Health Questionnaire 9 (Depression) compared to men, yet exhibited lower scores in Perceived Social Support and Resilience questionnaires. The age item showed differences in the Perceived Social Support Scale (PSSS) and the Resilience Scale. Participants aged 50–60 years reported higher scores on Perceived Social Support and Resilience Scales than other age groups. They also exhibited lower scores on Perceived Stress and Depression scales. The different levels of education exhibited differences in their scores on the Perceived Social Support Scale. Participants possessing higher educational attainments showed higher scores on the Perceived Stress Scale. However, the different kinds of job showed no significant differences on the four scales. The descriptive statistics for each variable are presented in Table 1.

**Table 1:** The descriptive statistics of perceived stress, perceived social support, resilience, and depression among COVID-19 patients^*^.

			Perceived stress					Perceived social support					Resilience					COVID-19 depression				
Characteristics	Categories	Fre	Mean	SD	Test	p	Effect size	Mean	SD	Test	p	Effect size	Mean	SD	Test	p	Effect size	Mean	SD	Test	p	Effect size
Gender^a^	Male	517	14.25	7.999	−3.775	0.000^***^	0.257^c^	57.57	17.201	2.131	0.033^*^	0.145^c^	24.56	10.492	2.86	0.004^**^	0.195^c^	6.3	5.606	−3.1	0.002^**^	0.211^c^
	Female	371	16.28	7.835				55.04	17.716				22.54	10.271				7.5	5.769			
Age^b^	18–30	302	15.18	7.252	0.503	0.734	0.002^d^	54.66	16.934	3.363	0.010^**^	0.015^d^	22.63	9.933	3.184	0.013^*^	0.014^d^	6.87	5.596	0.485	0.747	0.002^d^
	31–40	238	15.56	7.934				56.42	17.21				23.44	10.423				6.95	5.392			
	40–50	163	14.80	8.475				57.79	18.407				23.79	10.704				6.5	5.778			
	50–60	130	14.42	8.610				60.67	16.658				26.48	10.260				6.47	6.041			
	>60	55	15.15	9.178				53.49	18.724				24.15	11.988				7.51	6.588			
Education^b^	Junior high school and below	350	13.46	8.727	18.316	0.000^***^	0.040^d^	56.03	18.511	0.664	0.515	0.001^d^	23.6	10.814	0.29	0.749	0.001^d^	6.66	5.961	0.356	0.701	0.001^d^
	High school, technical school	364	15.38	7.701				56.34	16.981				24.02	10.388				6.8	5.702			
	Bachelor degree or above	174	17.81	6.02				57.85	16.222				23.33	9.819				7.1	5.164			
Job^b^	Freelance work	239	14.72	8.452	1.676	0.138	0.009^d^	56.30	17.131	0.34	0.889	0.002^d^	24.32	10.46	0.89	0.487	0.005^d^	6.86	6.047	0.192	0.966	0.001^d^
	farmer	4	15	6.218				53.75	10.308				24	6.164				7.5	1.732			
	staff	428	14.78	7.816				56.13	17.748				23.59	9.936				6.67	5.388			
	manager	86	17.2	7.877				58.55	18.538				21.74	11.558				7.29	6.056			
	student	51	16.29	6.392				57.59	15.954				24.67	10.539				6.86	6.177			
	retire	80	14.91	8.399				56.48	17.987				24.08	11.814				6.75	5.819			

^*^Fre, Frequency; Test, Test statistic; ^a^*t* test for independent group; ^b^Analysis of variance; ^c^Cohen’s d; ^d^eta-squared; p, *p* value; *p < 0.05, **p < 0.01, ***p < 0.001.

### 3.2 Pearson correlation analysis

To investigate the relationship between variables, we employed the Pearson correlation analysis. It showed a positive correlation between perceived stress and depression (*r* = 0.613, *p* < 0.01), indicating that higher perceived stress is associated with higher levels of depression. In contrast, the data showed that perceived social support and resilience scores were negatively correlated with depression (*r* = −0.521, *p* < 0.01; *r* = −0.527, *p* < 0.01). It showed that higher levels of perceived social support and resilience are associated with lower levels of depression. Furthermore, there was a negative correlation between perceived stress and perceived social support (*r* = −0.318, *p* < 0.01), resilience (*r* = −0.398, *p* < 0.01) which suggested that higher perceived stress is associated with lower levels of social support and resilience. Specific data are presented in Table 2.

**Table 2:** Bivariate correlations among main study variables.

	Scores	Perceived stress	Perceived social support	Resilience	Depression
	(Mean ± SD)				
Perceived stress	15.10 ± 7.99	–			
Perceived social support	56.51 ± 17.453	−0.318**	–		
Resilience	23.72 ± 10.443	−0.398**	0.460**	–	
Depression	6.80 ± 5.702	0.613**	−0.521**	−0.527**	–

**p < 0.01.

### 3.3 Mediation effects analysis

**Figure 2:**
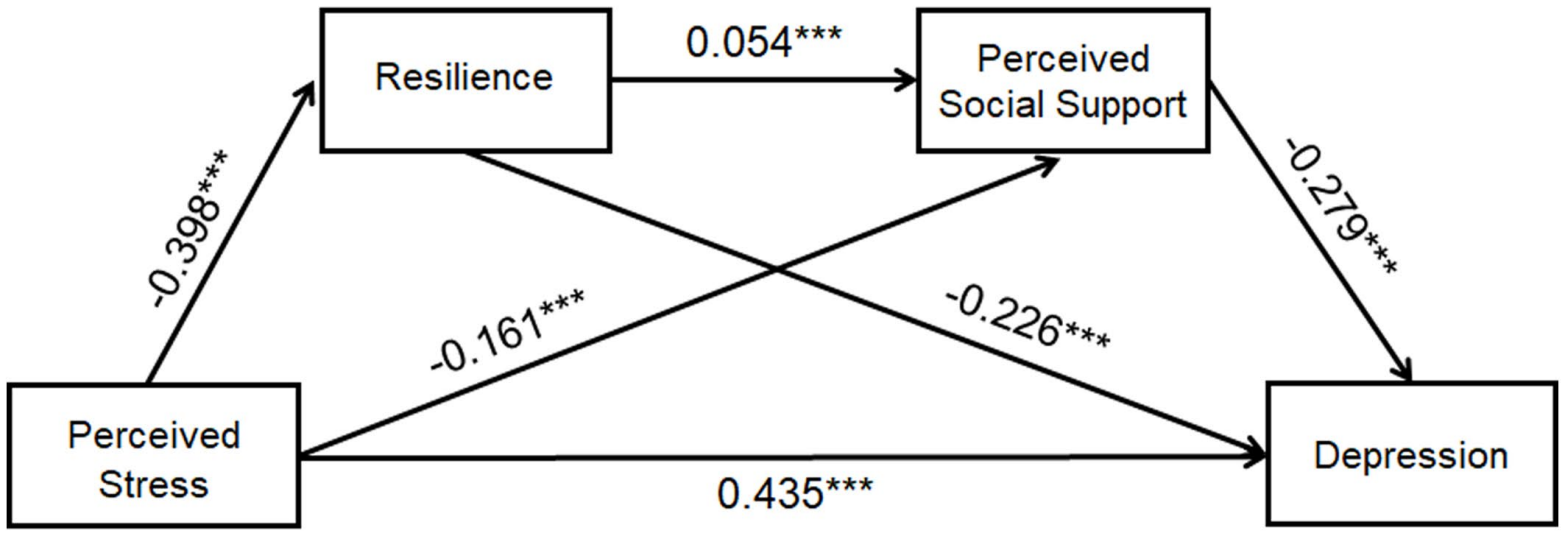
The path coefficient results of the chained mediation model between perceived stress, resilience, perceived social support and depression. Perceived stress can negatively predict resilience (*β* = −0.398) and perceived social support (*β* = −0.161), whereas positively predict depression (*β* = 0.435). Resilience and perceived social support were significant negative predictor of depression (*β* = −0.226, −0.279). Resilience was thought to be a significant positive predictor of perceived social support (*β* = 0.054). *p < 0.05, **p < 0.01, ***p < 0.001.

Model 6 of the SPSS plug-in process provided by Hayes was used (Hayes and Preacher, 2014). Perceived stress symptoms were taken as the independent variable, depression as the dependent variable, resilience and perceived social support as chained mediating variables. The path coefficient results are shown in Figure 2.

Table 3 lists the results of the multiple linear regression analysis of the major research variables. It also shows that perceived stress can significantly positively predict depression (*β* = 0.310, *p* < 0.001) and can significantly negatively predict resilience (*β* = −0.520, *p* < 0.001) and perceived social support (*β* = −0.352, *p* < 0.001). Resilience was thought to be a significant positive predictor of perceived social support (*β* = 0.661, *p* < 0.001) and a significant negative predictor of depression (*β* = −0.123, *p* < 0.001), while perceived social support was a significant negative predictor of depression (*β* = −0.091, *p* < 0.001).

**Table 3:** The relationship between perceived stress and depression: the chain mediating effect of perceived social support and resilience.

Items	Regression equation	Integral fitting index			coefficient		Significance of regression coefficient	
	Predictive variables	*R*	*R*^2^	*F*	*β*	standardization β	*t*	*P*
Resilience	Perceived Stress	0.398	0.158	166.554	−0.520^***^	−0.398	−12.906	0.000
Perceived Social Support	Perceived Stress	0.483	0.233	134.475	−0.352^***^	−0.161	−5.02	0.000
	Resilience				0.661^***^	0.054	12.327	0.000
Depression	Perceived Stress	0.729	0.531	333.525	0.310^***^	0.435	17.078	0.000
	Resilience				−0.123^***^	−0.226	−8.304	0.000
	Perceived Social Support				−0.091^***^	−0.279	−10.602	0.000

***p < 0.001.

The bootstrap test (Table 4) further showed that the 95% CI of the three paths did not include zero, indicating that the three indirect effects reached significance. Table 4 shows that the total effect of the relationship between perceived stress and depression was significant (effect = 0.4493, 95% CI: 0.0000, 0.4114). Meanwhile, the total indirect effect was significant (effect = 0.1328, 95% CI: 0.1013, 0.1703). The indirect effect was significant (effect = 0.0666, 95% CI: 0.0432, 0.0952) with resilience as the mediating variable. The indirect effect of the pathway with perceived social support as the mediating variable was significant (effect = 0.0354, 95% CI: 0.0163, 0.0603). The indirect effect of the pathway with resilience and perceived social support as mediating variables was also significant (effect = 0.0308, 95% CI: 0.0201, 0.0440).

**Table 4:** The mediating effect test of perceived social support and resilience^*^.

Item	Effect size	Boot SE	Boot CI		Proportion of indirect effect
			Lower	Upper	
Total effects	0.4493	0.0193	0.0000	0.4114	
Direct effect	0.3165	0.0187	0.0000	0.2798	
Total indirect effects	0.1328	0.0176	0.1013	0.1703	
Indp1: PS → RE → DE	0.0666	0.0132	0.0432	0.0952	14.82%
Indp2: PS → PSS → DE	0.0354	0.0111	0.0163	0.0603	7.87%
Indp3: PS → RE → PSS → DE	0.0308	0.061	0.0201	0.0440	6.86%

^*^Indp, indirect path; PS, perceived stress; RE, Resilience; PSS, perceived social support; DE, depression; CI, confidence interval.

## 4 Discussion

### 4.1 The chain mediating effects of resilience and perceived social support

The present study used a chained mediation model to investigate the relationship between perceived stress, resilience, perceived social support, and depressive symptoms among patients with COVID-19. This study found that perceived stress had a significant positive predictive effect on depression. Furthermore, the resilience and perceived social support had a chain mediating effect on the relationship between perceived stress and depression. The higher the level of perceived stress in COVID-19 patients, the lower their resilience level, which in turn led to lower perceived social support, thus led to higher depressive status. In the study, we found that perceived stress was a significant positive predictor of depression in COVID-19 patients, indicating that the higher the degree of perceived stress, the higher the level of depression, which is consistent with previous studies (Duan and Zhu, 2020; Damico et al., 2021). The relocation of COVID-19 patients to an isolated environment was a significant stressful event (Rauch et al., 2021), which can easily induce individual depression (Hammen, 2005). A stressor typically triggers a stress reaction if the stressor was perceived as a threat or demand, or the coping resources were perceived as insufficient for handling the given situation (Cohen et al., 1983). For COVID-19 patients, centralized isolation and treatment are required. Thus, they cannot go to work, seek support from loved ones and engage in their communities. Loneliness, fear of infection, suffering and death for oneself and for loved ones, grief after bereavement and financial worries have also all been cited as stressors leading to depression (Cottin et al., 2021). Hewitt and colleagues had highlighted the importance of perceived stress and coping resources in the prediction of stress-related symptoms (Hewitt et al., 1992). Therefore, it is necessary to recognize the negative influence of perceived stress on depression, and clarify the relationship between perceived stress and depression, which facilitates us to identify effective coping strategies to avoid mental health problems.

The results showed that perceived stress negatively predicted resilience, and resilience negatively predicted depression, which is consistent with other research results (Awano et al., 2020; Luceno-Moreno et al., 2020). Resilience had a mediating effect between perceived stress and depression, which verified the mediating path of resilience between perceived stress and depression. Resilience has been considered a personal characteristic that can help individuals preserve mental health in situations of severe stress or trauma (Connor, 2006; Poudel-Tandukar et al., 2019). It indicated better coping results in stressful situations (Burns et al., 2011). According to the stress buffer model of resilience (Richardson, 2002), the researchers found that resilience gradually varied with the way individuals cope with stress under stressful circumstances, and played an important role in the mitigation mechanism. Resilience can bring a positive transformation for individuals in adversity, greater threat, or trauma (Southwick and Charney, 2012). Some researchers have found that resilience minimized and buffered the negative influence of stress on mental health (Arrogante and Aparicio-Zaldivar, 2017). Furthermore, resilience was also considered as an index of mental health (Davydov et al., 2010). As a protective factor, resilience prompted individuals to change their original cognitive mode, mobilized personal resources, and coped with the stress flexibly, thus improving the mental health and reducing their depression level (Kalisch et al., 2017; Zhang et al., 2020; Kinser et al., 2021).

In addition, the results of the mediation analysis showed that perceived stress level significantly negatively predicted perceived social support, and perceived social support significantly negatively predicted depression level, indicating the mediating effect of perceived social support between perceived stress and depression. Perceived social support referred to an individual’s expectations, feelings and evaluation of social support situations (Wang et al., 1999). Social support referred to the support provided by family, friends and others as available to provide overall support (Xu et al., 2024). Research on relationships and health has pointed exclusively on the importance of supportive relationships in the context of stress or adversity (Cohen and Wills, 1985). Close relationships promoted well-being in many ways, not just as a resource in times of adversity. This can be explained by the following aspects. Firstly, when individuals faced stressful events, the perception of social support can produce positive mental states, including a sense of self-worth and a sense of security, which was beneficial to mental health and reduces the level of depression of individuals (Cohen, 2004). Secondly, perceived social support can also affect the regulation of the neuroendocrine response to stress and reduce the level of depression in individuals (Peng et al., 2022). Finally, the perception of social support can increase an individual’s sense of self-worth and self-control, generate positive evaluations of stressful events, and reduce their level of depression (Ozbay et al., 2007).

Moreover, this study found that perceived stress could have a significant influence on depression through the chain mediating effect of resilience and perceived social support, which verified the chain mediating path of resilience and perceived social support. This suggested that resilience not only acted as a single mediator between perceived stress and depression, but also further influenced depression by affecting perceived social support levels. The data showed that perceived social support was positively correlated with resilience, which is basically consistent with existing conclusions (Labrague and Santos, 2020). Feeney and Collins proposed an integrative model of thriving, in which perceived social support and resilience were thriving components (Feeney and Collins, 2015). This model pointed that perceived social support can provide emotional comfort and reassurance, convey understanding and acceptance, shield or defend the close other from negative forces related to the stressor, and benefit an individual’s ability to cope with stress, which is a catalyst for resilience.

Researchers have mostly regarded mental toughness as a process, that is, an individual’s ability to deal with adversity challenges, which has been proven to reduce the impact of traumatic events and the possibility of developing post-traumatic stress disorder (Lee et al., 2014). According to Kumpfer’s (1999) Resilience Framework, the selective perception was an internal mechanism that affected resilience. Some studies have pointed out that perceived social support (as a positive selective perception) was an important factor affecting resilience (Kalisch et al., 2015; Veer et al., 2021), which was based on the assumption that the belief that one can rely on others will make the potential stressors perceived as less threatening. Perceived social support can act as a protective factor against psychological resilience (Masten and Garmezy, 1985; Werner, 2004). Studies have suggested that perceived social support mediated the relationship between mental resilience and burden (Ong et al., 2018). Previous studies showed high levels of social support and resilience were protective factors against alcohol and drug abuse amongst the general public (Studer et al., 2017; Caravaca-Sánchez and García-Jarillo, 2020). According to the study, it is necessary to pay attention to the physical and mental conditions of COVD19 patients during clinical work. At the same time, the perceived social support of patients needs to be evaluated to improve resilience levels, thus reducing depression in COVID-19 patients with stress.

### 4.2 Limitations and future research

While this study has provided valuable insights, there are some limitations to this study as well. Firstly, the study was confined to a single hospital with a centralized COVID-19 patient management system. This may introduce bias as management systems and work schedules at other hospitals could potentially influence the variables. To validate the findings, future studies should extend their scope to multiple regions and hospitals. Secondly, the research was limited to perceived stress and depression among COVID-19 patients, ignoring other mental health outcomes such as anxiety. Nevertheless, we postulate that the relationships uncovered in this study likely extend to other conditions.

## 5 Conclusion

Based on the above, the present model shows that perceived stress influences depression through the independent and combined effects of two mediating variables, namely perceived social support and resilience, which further explains the mechanism of the influence of perceived stress on depression, as well as provides new perspectives for the relationship between the perceived stress and depression for COVID-19 patients. In the future, when suffering public health crises such as pandemics, we could attempt to mobilize individuals’ positive psychological traits to improve their mental health in clinical practice. This finding confirms and extends the protective effect of perceived social support and resilience that are being assessed in populations of adults confronted with the epidemic crisis situations.

## Data Availability

The original contributions presented in the study are included in the article/supplementary material, further inquiries can be directed to the corresponding authors.

## Footnotes

^1^ www.wenjuan.com
